# A Repurposed Drug Screen Identifies Compounds That Inhibit the Binding of the COVID-19 Spike Protein to ACE2

**DOI:** 10.3389/fphar.2021.685308

**Published:** 2021-06-14

**Authors:** Kaleb B. Tsegay, Christiana M. Adeyemi, Edward P. Gniffke, D. Noah Sather, John K. Walker, Stephen E. P. Smith

**Affiliations:** ^1^ Center for Integrative Brain Research, Seattle Children’s Research Institute, Seattle, WA, United States; ^2^ St. Louis University School of Medicine, Department of Pharmacology and Physiology, St. Louis, MO, United States; ^3^ Center for Global Infectious Disease Research, Seattle Children’s Research Institute, Seattle, WA, United States; ^4^ Department of Pediatrics, University of Washington, Seattle, WA, United States; ^5^ Henry and Amelia Nasrallah Center for Neuroscience, Saint Louis University St. Louis, Seattle, WA, United States; ^6^ Graduate Program in Neuroscience, University of Washington, Seattle, WA, United States

**Keywords:** COVID-19, drug screen, IP-FCM, inhibition assay, repurposed

## Abstract

Repurposed drugs that block the interaction between the SARS-CoV-2 spike protein and its receptor ACE2 could offer a rapid route to novel COVID-19 treatments or prophylactics. Here, we screened 2,701 compounds from a commercial library of drugs approved by international regulatory agencies for their ability to inhibit the binding of recombinant, trimeric SARS-CoV-2 spike protein to recombinant human ACE2. We identified 56 compounds that inhibited binding in a concentration-dependent manner, measured the IC_50_ of binding inhibition, and computationally modeled the docking of the best inhibitors to the Spike-ACE2 binding interface. The best candidates were Thiostrepton, Oxytocin, Nilotinib, and Hydroxycamptothecin with IC50’s in the 4–9 μM range. These results highlight an effective screening approach to identify compounds capable of disrupting the Spike-ACE2 interaction, as well as identify several potential inhibitors of the Spike-ACE2 interaction.

## Introduction

COVID-19 is currently a global pandemic, causing extensive mortality and economic impact. While the success of rapidly developed vaccines offers hope to control the virus (Polack et al., 2020), treatments that improve disease outcomes are also critically needed. Dexamethasone, an anti-inflammatory steroid, is FDA-approved to treat COVID-19, as is Remdesivir, a nucleoside analogue prodrug that inhibits viral RNA polymerase (Beigel et al., 2020), though its efficacy is disputed (WHO Solidarity Trial Consortium, 2021). Blocking the interaction between the Severe Acute Respiratory Syndrome Corona Virus 2 (SARS-CoV-2) spike protein and its obligatory receptor Angiotensin-converting enzyme 2 (ACE2), has also shown promise as a therapy; recombinant soluble ACE2 is effective in a cell culture model (Monteil et al., 2020), and three different monoclonal antibody drugs are now FDA approved (Marovich et al., 2020). However, these biologic drugs are expensive and suffer production limitations. Repurposing already-approved small molecule drugs, particularly those that might block the interaction between ACE2 and spike, could allow for rapid deployment of low-cost and widely available therapeutics (Saul and Einav, 2020). Thus far, all repurposed drug candidates have failed to reduce mortality, initiation of ventilation or hospitalization duration in robust clinical trials (Cao et al., 2020; BJM, 2020; WHO Solidarity Trial Consortium, 2021); however none of these candidates act trough a mechanism that involves blocking the ACE2-spike interaction.

Here, we aimed to identify repurposed drugs that could block the interaction between the SARS-CoV-2 spike protein and ACE2. We screened 2,701 drugs approved by global regulatory agencies using a previously published assay (Gniffke et al., 2020) that measures inhibition of binding between the trimeric SARS-CoV-2 spike protein (Wrapp et al., 2020) and latex-bead-conjugated recombinant human ACE2. We identified 56 compounds that inhibited the spike-ACE2 interaction by < 90% at 1 mM and that produced dilution curves that yielded an IC_50_ value, and further characterized the 12 compounds with the lowest half-maximal inhibitory concentration (IC_50_) using in silico modeling of the compounds’ interaction with the binding interface.

## Methods

### Drug Screening

The “FDA-approved drug screening library” (Cat #L1300) was purchased from Selleck Chemicals. Recombinant ACE2 and trimeric spike protein were produced in-house using previously published protocols (Gniffke et al., 2020). In a 96-well plate format, we briefly incubated recombinant, biotinylated, trimeric spike protein (Wrapp et al., 2020) with either 200 µM or 1 mM of each drug, in duplicate, then added 5-micron flow cytometry beads (Luminex) coated with recombinant ACE2 [for detailed methods, see (Gniffke et al., 2020)]. Three replicates per plate of positive (vehicle) controls and negative no-spike-protein controls were included, 31 plates in total. After washing and the addition of streptavidin-PE to bind spike attached to ACE2, plates were washed again on a magnetic plate washer and read on an Acea Novocyte flow cytometer. Data were expressed as the median PE fluorescence intensity (MFI), and converted to % inhibition using the formula 1-(MFI_drug_/MFI_positive control_). For IC_50_ studies, two independent serial dilutions of drugs were performed and run as above. IC_50_ was calculated in Graphpad Prism using the Hill equation for a normalized response with variable slope (four parameter).

### In Silico Modeling

Docking experiments were performed with the SARS-COV-2 spike protein (PDB ID: 6VSB) and ACE2 (PDB ID: 2AJF). The region selected for docking studies was the receptor binding domain (RBD) of the Spike protein and the corresponding region of ACE2, with docking grids generated around key binding residues at the Spike-ACE2 interface. The missing loops in the S1 subunit of the 6VSB structure, which contains the RBD, were reconstructed using the SWISS-MODEL server (Waterhouse et al., 2018). The FASTA sequence (residues 316-530) was retrieved from UniProtKB-P0DTC2 and used as a query sequence, PDB ID: 6VSB with 100% sequence identity was used as a template (Berman et al., 2000; Pundir et al., 2016). The structure quality of the modelled protein was validated on PROCHECK, which showed 89.2% residues in the core regions, the quality factor of 89.77% was obtained from Verify 3D on the SAVES server and ProSA web gave a Z-score of-6.15 (Laskowski et al., 1993; Eisenberg et al., 1997; Wiederstein and Sippl, 2007). The crystal structure of ACE2 was retrieved from PDB 1D: 2AJF, pre-processed with Prime on Schrödinger, and used for docking into the RBD interface. The SDF structures of the selected FDA-approved drugs were downloaded from Selleck chemicals and PubChem. Ligand and protein preparation was performed using the Ligprep and protein preparation wizard tool on Schrödinger Maestro version 12.2. Structural-based docking to the RBD interface of the Spike and ACE2 was also performed using Schrödinger Maestro and BIOVIA Discovery Studio (Dassault Systems) for docking analysis and visualization.

## Results

**FIGURE 1:**
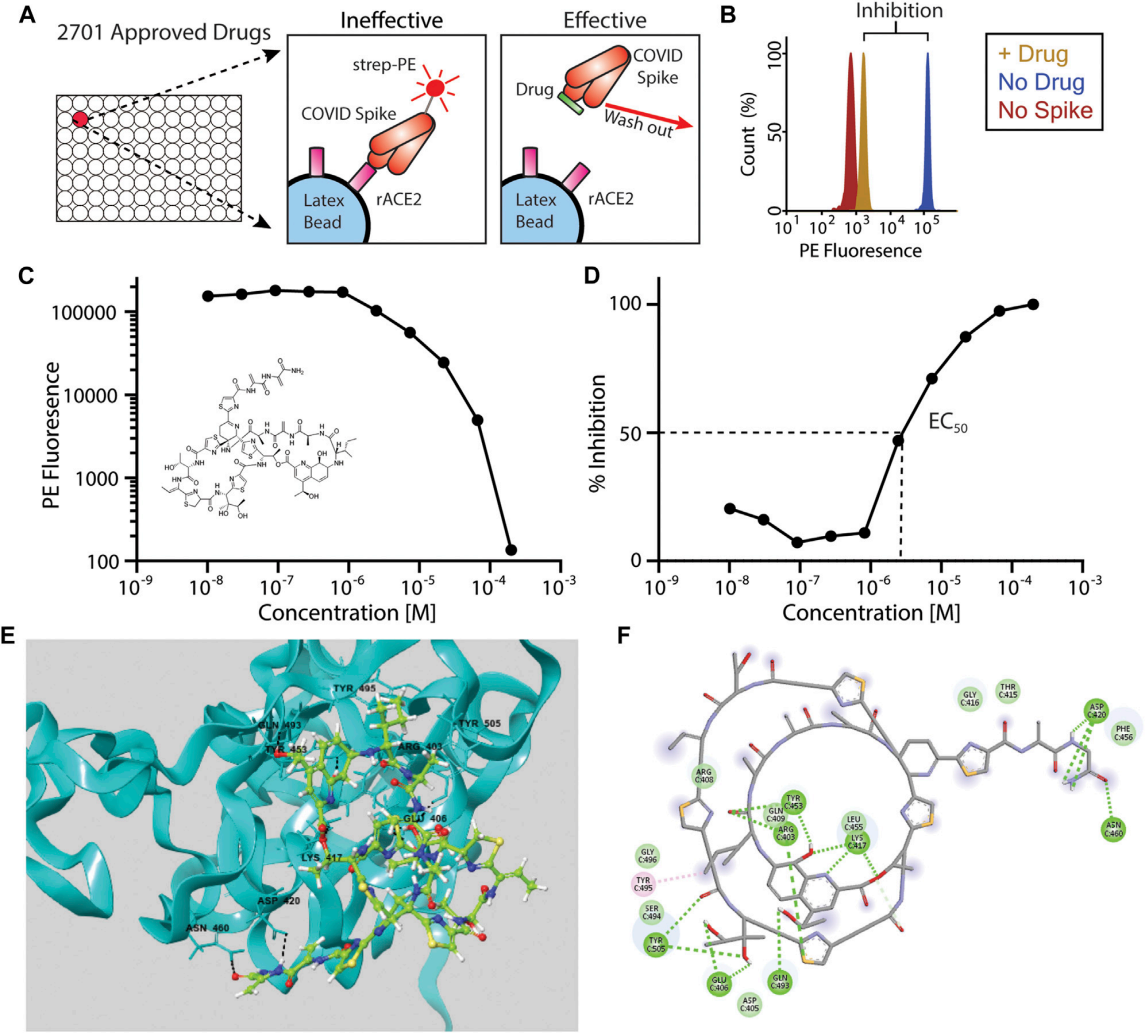
Inhibition of Spike-ACE2 binding by repurposed drugs. **(A)** *In vitro* assay design showing inhibition of ACE2-Spike binding by “effective” drugs. Note that compounds may also bind to ACE2, in addition to the spike protein as illustrated here. **(B)** Example histogram from primary screen showing >90% inhibition of ACE2-Spike binding following drug addition. **(C, D)** IC_50_ data for the top candidate, Thiostrepton (structure inset) expressed as PE fluorescence **(C)** or percent inhibition **(D)**. **(E)** Three-dimensional and **(F)** two-dimensional computational rendering of Thiostrepton binding to the SARS-CoV-2 spike protein.

We took an unbiased approach to screen 2,701 drugs approved by global regulatory agencies for the ability to block the interaction between recombinant, trimeric SARS2 spike protein (Wrapp et al., 2020), and latex-bead-conjugated recombinant human ACE2 using a previously published assay (Gniffke et al., 2020) (Figure 1A). The use of a cell-free system prevented potential cytotoxic effects of drugs inherent to cell-culture-based, live-virus assays, and allowed us to focus solely on inhibition of the Spike-ACE2 interaction. We quantified the amount of Spike-ACE2 co-association in the presence of high concentrations (200 µM–1 mM) of each drug, in duplicate, and calculated the percent inhibition using six replicates of vehicle control per plate (31 plates total). In this first-round screen, 114 drugs that exhibited 90% or greater inhibition were identified (Figure 1B; Supplementary Table S1).

We next performed serial dilutions of these 114 drugs to measure the IC_50_s of the ACE2–spike interaction (Figures 1C,D). Fifty-eight of the drugs were revealed to be either false positive hits (they showed no inhibition upon re-screening), or showed inhibition only at the highest concentration tested, and were eliminated. The drug with the lowest (best) IC_50_ was Thiostrepton (IC_50_ = 3.95 ± 0.02 x 10^−6^ M), a cyclic oligopeptide used as a topical antibiotic in animals that interacts with the transcription factor FOXM1 to inhibit the growth of breast cancer cells *in vitro* (Hegde et al., 2011). Next was Oxytocin (IC_50_ = 4.10 ± 0.07 x 10^−6^ M), a peptide hormone that is administered to induce childbirth, and that may increase social cognition when administered intranasally (Keech et al., 2018). The next four best candidates were actually two closely related pairs of drugs, which demonstrates the robustness of our screen in identifying each compound twice. Nilotinib, which was identified as both a free base (IC_50_ = 4.21 ± 0.36 × 10^−6^ M) and an HCl salt (IC_50_ = 8.43 ± 1.18 × 10^−6^ M), is a selective tyrosine kinase inhibitor used to treat chronic myelogenous leukemia (Tokuhira et al., 2018). Hydroxycamptothecine (IC_50_ = 6.87 ± 0.77 × 10^−6^ M) and its stereoisomer S-10-Hydroxycamptothecine (IC_50_ = 7.22 ± 0.06 × 10^−6^ M) are DNA topoisomerase I inhibitors with anti-cancer activity (Fei et al., 2013). Interestingly, three derivatives that have also been approved for cancer therapy, Topotecan, Irinotecan, and Belotecan were included in the screening panel, but did not inhibit spike-ACE2 binding. The IC_50_s of all 56 compounds are listed in Supplementary Table S2; given the decreased severity of COVID-19 in females (Peckham et al., 2020), it is notable that Estradiol Benzoate (a synthetic estrogen) inhibited the interaction (IC_50_ = 1.75 × 10^−5^ M), although the IC50 we measured is far greater than the physiological concentration of estrogen.

We next performed molecular docking studies on the top twelve screening hits with both the Spike and ACE2 proteins, focusing on the interface region between the receptor binding domain (RBD) of the Spike protein and ACE2, to provide mechanistic insight into our identified compounds’ inhibitory activity. The Cryo-EM structure (PDB ID: 6VSB) was used for docking since the bioassay used the same trimeric spike protein plasmid construct as was used in determining that structure. The two most active screening hits, thiostrepton and oxytocin, were predicted to bind preferentially to the Spike protein based upon their Glide scores (Table 1), interacting with several key residues that mediate Spike-ACE2 binding (Hegde et al., 2011) (Figure 1; Supplementary Figures S1, S2; Supplementary Table S3). Thiostrepton in particular appears to bind extensively to Spike residues, but the OH-group in Thiostrepton was found to bind simultaneously with Lys417 of Spike and Asp30 of ACE2. Simultaneous binding of these two critical interface resides would likely disrupt the Spike-ACE2 interaction, resulting in the low observed EC50 value. Nilotinib and Hydroxycamptothecine exhibited higher Glide scores than thiostrepton and oxytocin in the Spike protein, but still within reasonable ranges. They were, however, predicted to have slightly more favorable binding with ACE2. Interestingly, both interacted with Arg393 on ACE2, a critical spike-binding residue, and both also interacted with the Spike receptor binding interface (Supplementary Figures S3–S6; Supplementary Table S3). The remaining compounds generally gave poorer glide scores, especially in the Spike protein. Docetaxel, Anidulafungin, and Estradiol, however, gave Glide scores in ACE2 that were comparable to the hydroxycamptothecin analogs, suggesting they may bind there. Some of the compounds such as Selamectin, Picropodophyllin, and Doramectin were predicted to bind poorly to both Spike and ACE2 (see Supplementary Figures S7–S12; Supplementary Table S3, and Supplementary Discussion). It is possible that these compounds bind to either Spike or ACE2 in a non-competitive manner outside the interface region that could result in conformational changes to the protein and thus disrupt the Spike-ACE2 interaction, as has been suggested for estradiol benzoate (Yang et al., 2021).

**TABLE 1:** Summary of the top 12 drug candidates. Computationally modeled glide scores for ACE2 and spike binding and IC_50_ values measured with the recombinant Spike-ACE2 binding inhibition assay are displayed.

	IC_50_		Glide score		Prior evidence:
	Mean	St.Dev	Spike	Ace2	
Thiostrepton	3.95E-06	2.19E-08	−7.173	−4.819	-
Oxytocin	4.10E-06	7.14E-08	−7.024	−5.205	Computational Mamkulathil Devasia et al. (2021)
Nilotinib AMN-107	4.21E-06	3.66E-07	−5.669	−6.207	Cell culture inhibition Fan et al. (2020), computational Zhang et al. (2021)
Hydroxycamptothecin	6.87E-06	7.71E-07	−5.321	−5.679	Computational Ahsan and Sajib (2021)
S-(10)-hydroxycamptothecin	7.22E-06	5.52E-08	−5.335	−5.673	*see Hydroxycamptothecin*
Nilotinib HCl	8.43E-06	1.18E-06	−5.659	−6.209	*See Nilotinib AMN-107*
Selamectin	8.47E-06	3.68E-08	−4.207	−3.503	Cell culture inhibition Hanson et al. (2020)
Picropodophyllin	9.84E-06	3.99E-06	−4.072	−4.158	
Docetaxel	1.01E-05	1.66E-06	−4.737	−5.359	
Doramectin	1.28E-05	1.06E-07	−4.65	−3.964	Computational Leake et al. (1980)
Anidulafungin	1.32E-05	1.35E-06	−4.649	−5.846	Computational Zhang et al. (1998)
Estradiol benzoate	1.74E-05	5.11E-06	−4.003	−5.345	Cell culture inhibition Dyall et al. (2014), clinical Bojadzic et al. (2020)

## Discussion

Overall, this study identified 56 approved drugs that show some efficacy in blocking the interaction between the COVID spike protein and its receptor, ACE2. Many of the identified drugs are already approved for clinical use in humans (Table 2). Moreover, several of the identified drugs have already shown promising results in computational modeling or cell-based studies (Table 1; Supplementary Table S2). Nilotinib (Hit #3 and #6 Table 1) has been reported to inhibit SARS-CoV-2 infection *in vitro* with an IC_50_ of 1.5–3 µM (Cagno et al., 2021), similar to our IC50 of 4 µM. The related Abl tyrosine kinase inhibitors Dasatinib and Imatinib were reported to inhibit SARS-CoV-1 and Middle Eastern Respiratory Syndroms (MERS) coronaviruses (Dyall et al., 2014). In our screen, three different formulations of Dasatinib inhibited > 90% in the first round, but only Dasatinib HCl (Hit #40 Supplementary Table S2) yielded an IC_50_ value, and Imatinib failed to inhibit in the first round screen. Similarly, Dasatinib, and Imatinib failed to inhibit SARS-CoV-2 in cell-culture-based studies (Cagno et al., 2021). Selamectin (Hit #7, Table 1) was one of three compounds identified in a screen of 2,406 clinically approved drugs that used a SARS-CoV-2-related pangolin corona virus in cell culture (Fan et al., 2020). This study also identified Cepharanthine (Hit #42 Supplementary Table S2), and Mefloquine (failed round 1, with 77% inhibition). Estradiol benzoate (Hit #12 Table 1) has also been reported to inhibit SARS-COV-2 replication in culture (Yang et al., 2021), possibly *via* binding to Spike in an area outside of the RBD. In humans, a retrospective study of women over 50 years old taking hormone therapy showed a 50% reduction in COVID-19 fatality that was not present in women 15–49 years old (Seeland et al., 2020). However, estrogen has a wide range of effects, including anti-inflammatory effects, and the concentration of estrogen *in vivo* is orders of magnitude less than that our observed IC_50_, so these data should be interpreted with caution.

**TABLE 2:** Reported pharmacokinetic properties of top hits. All studies were performed on humans unless otherwise indicated.

	**Indication**	**C**_**max**_ **(ng/ml)**	**MW (g/mol)**	**C**_**max**_ **(uM)**	**EC**_**50**_ **(uM)**	**Elimination T**_**1/2**_	**Dosing Regimen**	**References**
Thiostrepton	Topical antibiotic, vetrinary		1,664		4.0			
Oxytocin	Modify social behavior (expeirmental)	0.005	1,007	0.005	4.2	>1 h	Intranasal Single dose, 44 ug	Gossen et al (2012)
	Induction of labor	0.005	1,007	0.005	4.2	>>1 h	IV infusion 6.7 ng/min	Leake et al. (1980)
Nilotinib	Kinase inhibitor, Cancer treatment	1,360	529	2.57	4.2	16 h	Oral BID 300 mg	Tian et al. (2018)
Hydroxycamptothecin (in Rats)		15,930	364.4	43.7	7.3	428 min	RATS 10 mg/kg IV	Zhang (1998)
Selamectin (in Dogs)		86.5	770	0.11	8.5	266 h	DOGS topical 24 mg/kg	Sarasola et al. (2002)
		7,630	770	9.9	8.5	45.7 h	DOGS oral 24 mg/kg	Sarasola et al. (2002)
Picropodophyllin (PPP)	IGF inhibitror, Cancer treatment (aka AXL1717)	207–1,035	414	0.5–2.5	10.0	2 h	Oral 390 or 520 mg BID	Ekman et al. (2016)
Docetaxel	Microtubule inhibitor, Cancer treatment	933	808	1.23	10.3	25.4 h	IV infusion 30–36 mg/m^2^	Gustafson et al. (2003)
Doramectin	Antiparasitic, vetrinary	12.2	899	0.0135	12.8	10 days	CATTLE topical 500 ug/kg	Gayrard et al. (1999)
Anidulafungin	Antifungal	2,500	1,140	2.2	13.1	27 h	IV infusion 100 mg	Wasmann et al. (2018)
Estradiol Benzoate	Contraceptive	0.75	376	0.002	17.3	3 days	IM injection, 5 mg	Oriowo et al. (1980)

We also note that at least two additional studies have screened compounds for the ability to block the receptor binding domain (RBD) of the Spike protein from binding to ACE2. Hanson et al.(2020) identified a single confirmed hit, Corilagin, which was not included in our drug panel. A second study (Bojadzic et al., 2020) screening organic dyes identified Methylene Blue as a potential inhibitor, which failed our screen in the first round (44% inhibition). Methodological differences, as well as our use of a stabilized prefusion trimeric spike protein, which behaves differently from the smaller RBD construct in our assay (Gniffke et al., 2020), may account for the differences between these studies.

Aiming towards clinical translation, there are several obvious issues with our identified compounds. First, many of the drugs are chemotherapy agents, and have toxic side-effects that would not be tolerable in COVID-19 patients. For example, Nilotinib inhibits a kinase important in B cell signaling (Tian et al., 2018; Tokuhira et al., 2018), and may prevent normal immune function, while Picropodophyllin (Ekman et al., 2016) and Docetaxel (Gustafson et al., 2003) both produce moderate to severe side effects when used in the context of chemotherapy. Secondly, several of the drugs have peak plasma concentrations that are orders of magnitude lower than the concentration required to inhibit spike-Ace2 binding in the *in vitro* assay, for example Oxytocin (Gossen et al., 2012) and Doramectin (Gayrard et al., 1999) (0.005 vs 4.2 µM and 0.014 vs 12.8 µM, respectively). Finally, Oxytocin’s clearance rate would necessitate continuous infusion, which seems impractical.

In light of known side-effects and pharmacokinetic data of many of our high-ranking drug candidates, Selamectin (Hit #7, Table 1) may be the top candidate for further study. Selamectin is an anti-parasitic used in dogs and cats to prevent infestation with nematode and arthropod species, and is structurally related to Ivermectin. Oral Selamectin is well tolerated in dogs and can achieve a peak plasma concentrations (9.9 µM) (Sarasola et al., 2002) comparable to both our measured IC_50_ (8.5 µM), and the 10 µM dose that inhibited the cytopathic effect of a SARS-CoV-2-related Pangolin corona virus on Vero E6 cells in culture (Fan et al., 2020). However, we were not able to identify any studies using Selamectin in humans, since Ivermectin is the standard alternative. Given recent controversy over Ivermectin as a treatment for COVID-19 (Ivermectin (2021)), it is worth noting that a different Ivermectin derivative, Doramectin, was our #10 hit, and Ivermectin itself just missed our first round cut-off with 89% binding inhibition. Thiostrepton may also be a top candidate, but pharmacokinetic data are lacking.

An advantage of our recombinant approach, using a cell-free system, is that the effects of drug toxicity on cell growth do not confound the readout of Spike-Ace2 binding. However, our results would need to be replicated in a cell or animal model using live virus to ensure that anti-SARS-CoV-2 effects appear at drug concentrations low enough to prevent toxicity. In addition, we did not confirm if the compounds bound directly to ACE2 or spike protein, only that their presence inhibited the binding of the two proteins. The relatively weak (micromolar) binding kinetics of the drugs identified here, as well as their known toxicities, bioactivities and/or high clearance rates, suggest that many would currently be unlikely to be viable for treating acute disease or for prophylactic use. However, they could serve as starting points for future medicinal chemistry optimization efforts to rationally design derivatives that are both less toxic and bind to the COVID spike with higher affinity.

## Data Availability

The original contributions presented in the study are included in the article/Supplementary Material, further inquiries can be directed to the corresponding author.
