# Supplementary material for: A Repurposed Drug Screen Identifies Compounds That Inhibit the Binding of the COVID-19 Spike Protein to ACE2
Source: Front Pharmacol. 2021 Jun 14;12:685308. doi: 10.3389/fphar.2021.685308 (PMC8236845; doi:10.3389/fphar.2021.685308)
Supplement: Supplementary file 1 [file DataSheet2.PDF]

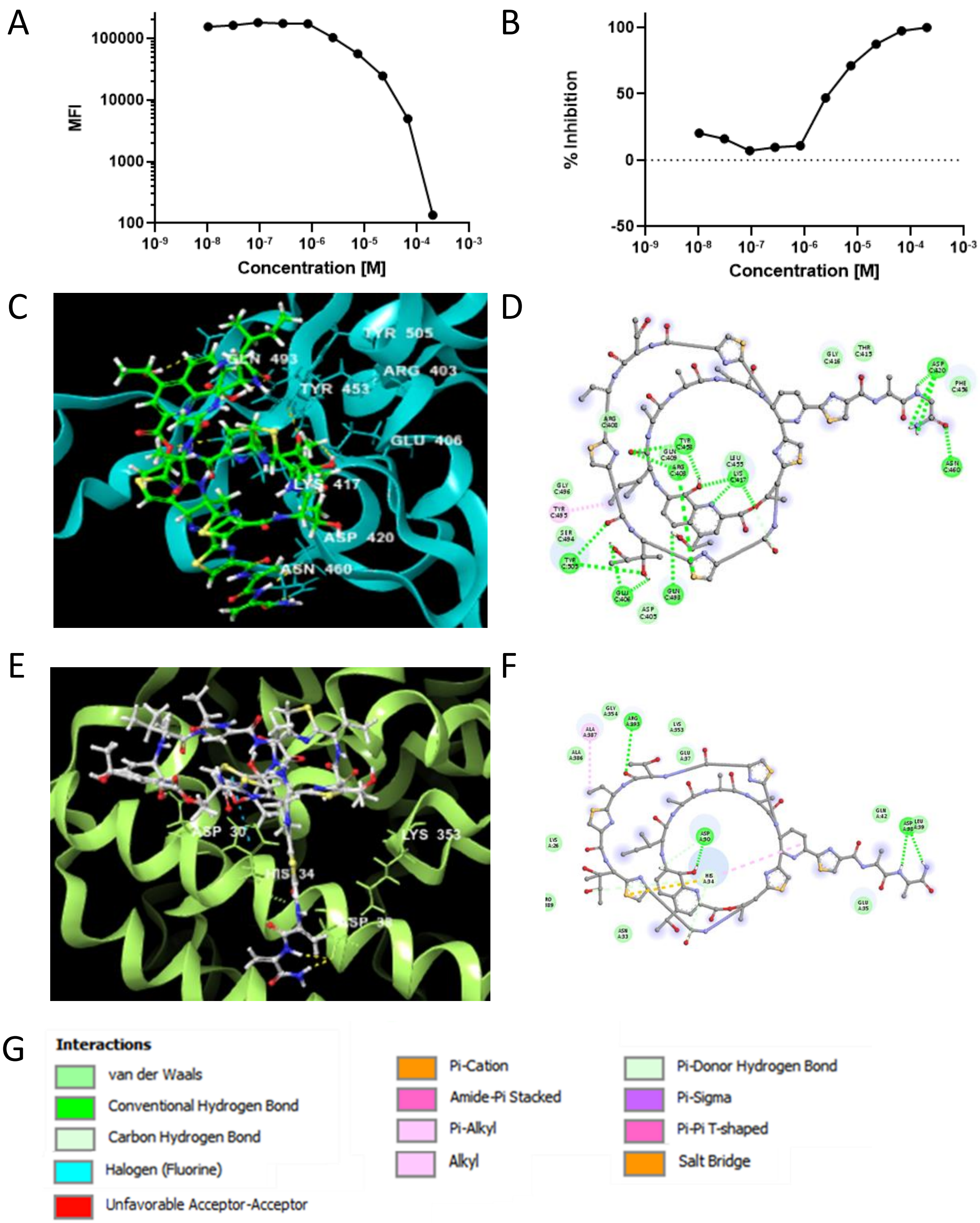

Figure S1: EC50 and computational binding data for Thiostrepton. A,B) EC50 data are expressed as median fluorescent intensity (MFI, A) or percent inhibition (B). C) Three dimensional and D) two dimensional computationally determined lowest-energy docking poses of Thiostrepton and the SARS-CoV-2 spike protein. E) Three dimensional and F) two dimensional computational docking of Thiostrepton and human ACE2. G) Key showing color-codes for interaction types. Note A-D are identical to Figure 1 C-F.

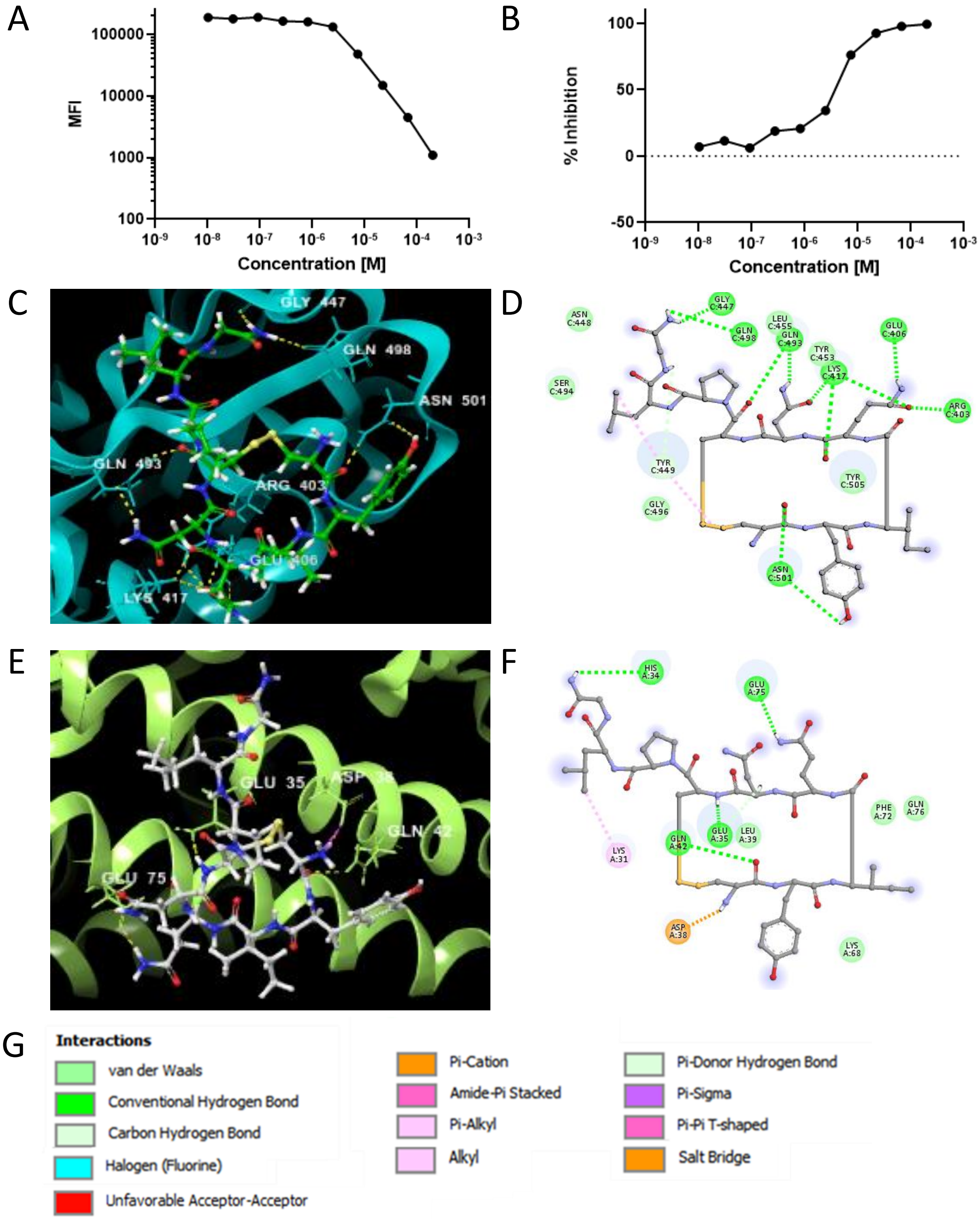

Figure S2: EC50 and computational binding data for Oxytocin. A,B) EC50 data are expressed as median fluorescent intensity (MFI, A) or percent inhibition (B). C) Three dimensional and D) two dimensional computationally determined lowest-energy docking poses of Oxytocin and the SARS-CoV-2 spike protein. E) Three dimensional and F) two dimensional computational docking of Oxytocin and human ACE2. G) Key showing color-codes for interaction types.

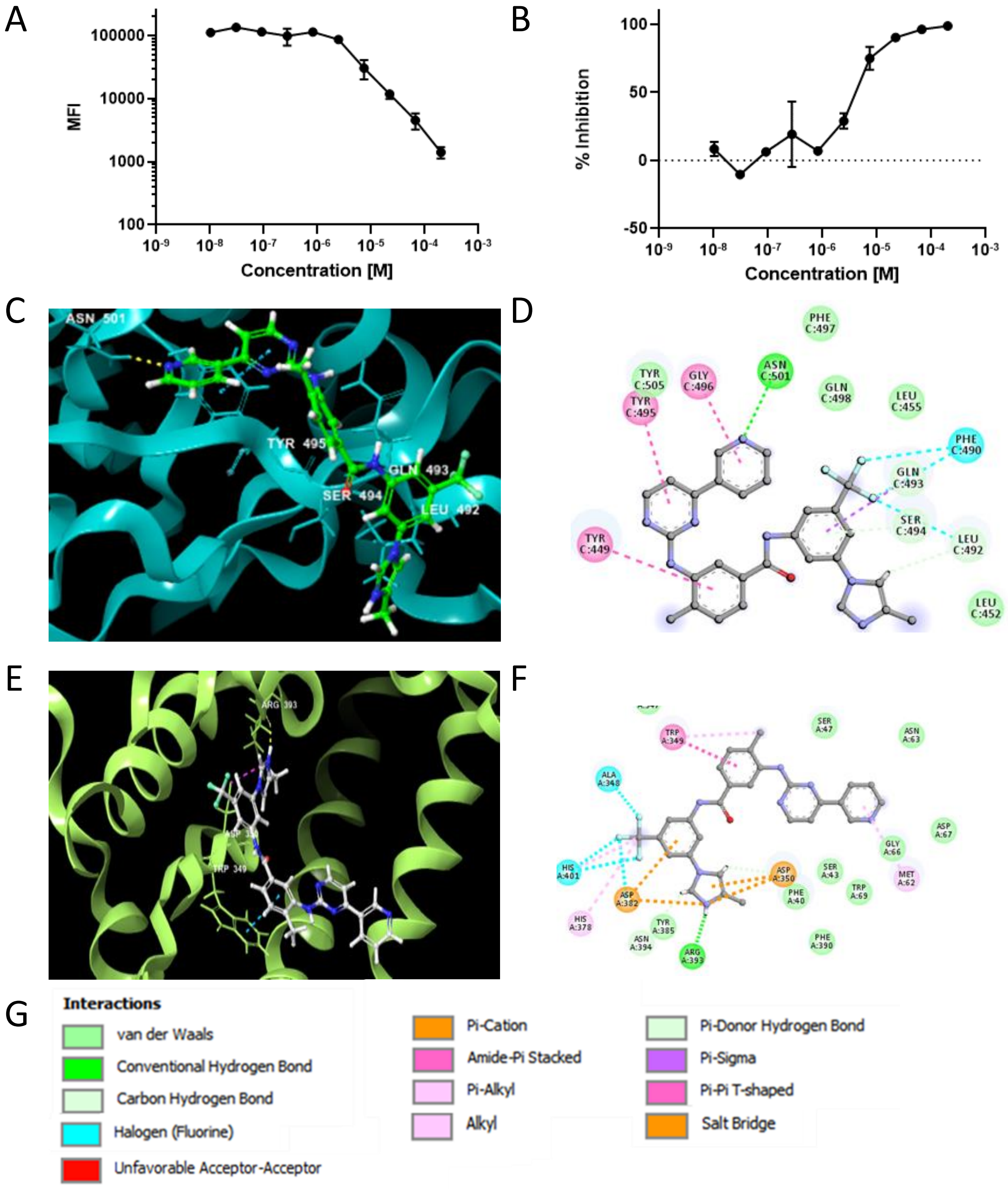

Figure S3: EC50 and computational binding data for Nilotinib (AMN-107). A,B) EC50 data are expressed as median fluorescent intensity (MFI, A) or percent inhibition (B). C) Three dimensional and D) two dimensional computationally determined lowest-energy docking poses of Nilotinib and the SARS-CoV-2 spike protein. E) Three dimensional and F) two dimensional computational docking of Nilotinib and human ACE2. G) Key showing color-codes for interaction types.

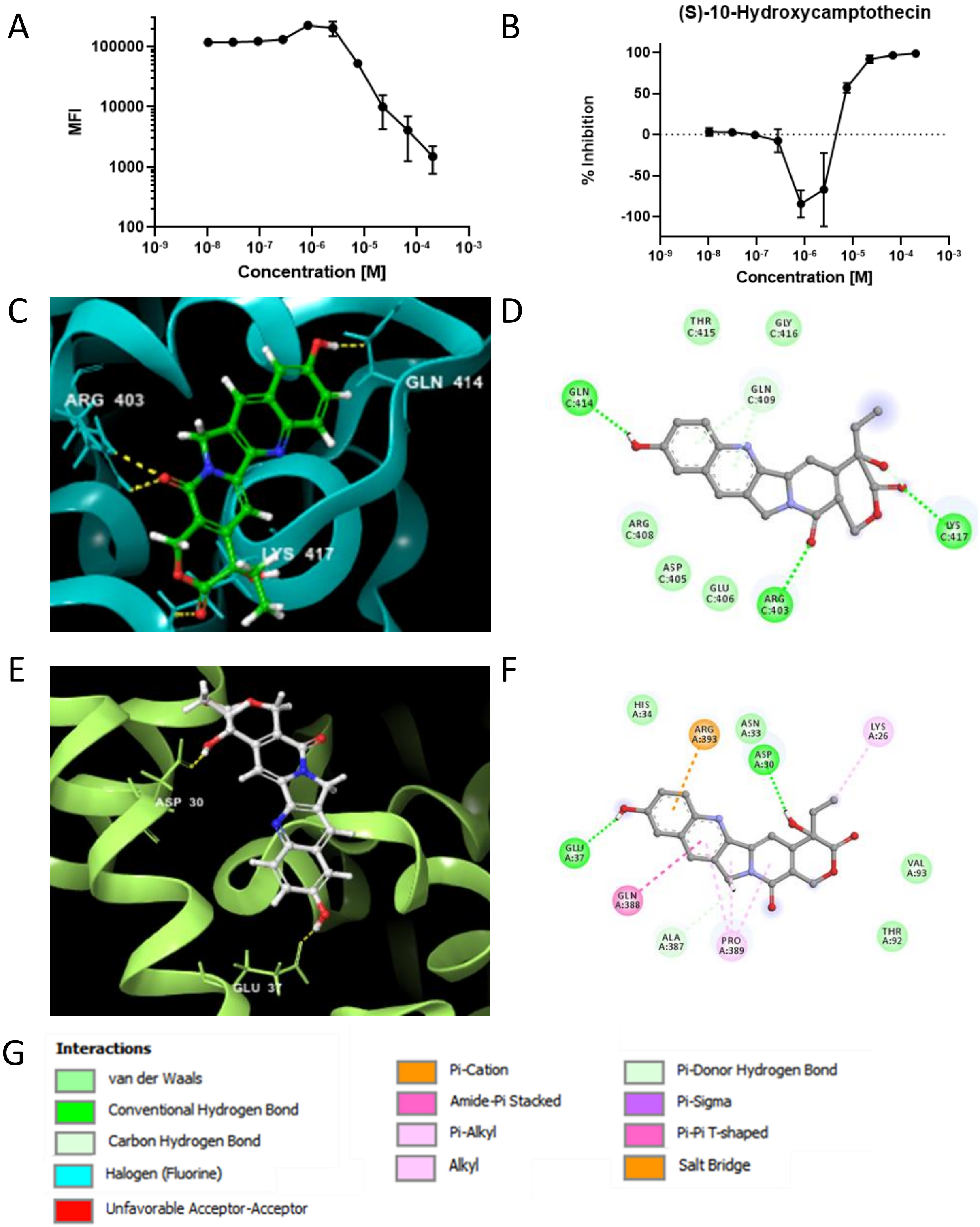

Figure S4: EC50 and computational binding data for (S)-10-Hydroxycamptothecin. A,B) EC50 data are expressed as median fluorescent intensity (MFI, A) or percent inhibition (B). C) Three dimensional and D) two dimensional computationally determined lowest-energy docking poses of (S)-10-Hydroxycamptothecin and the SARS-CoV-2 spike protein. E) Three dimensional and F) two dimensional computational docking of (S)-10-Hydroxycamptothecin and human ACE2. G) Key showing color-codes for interaction types.

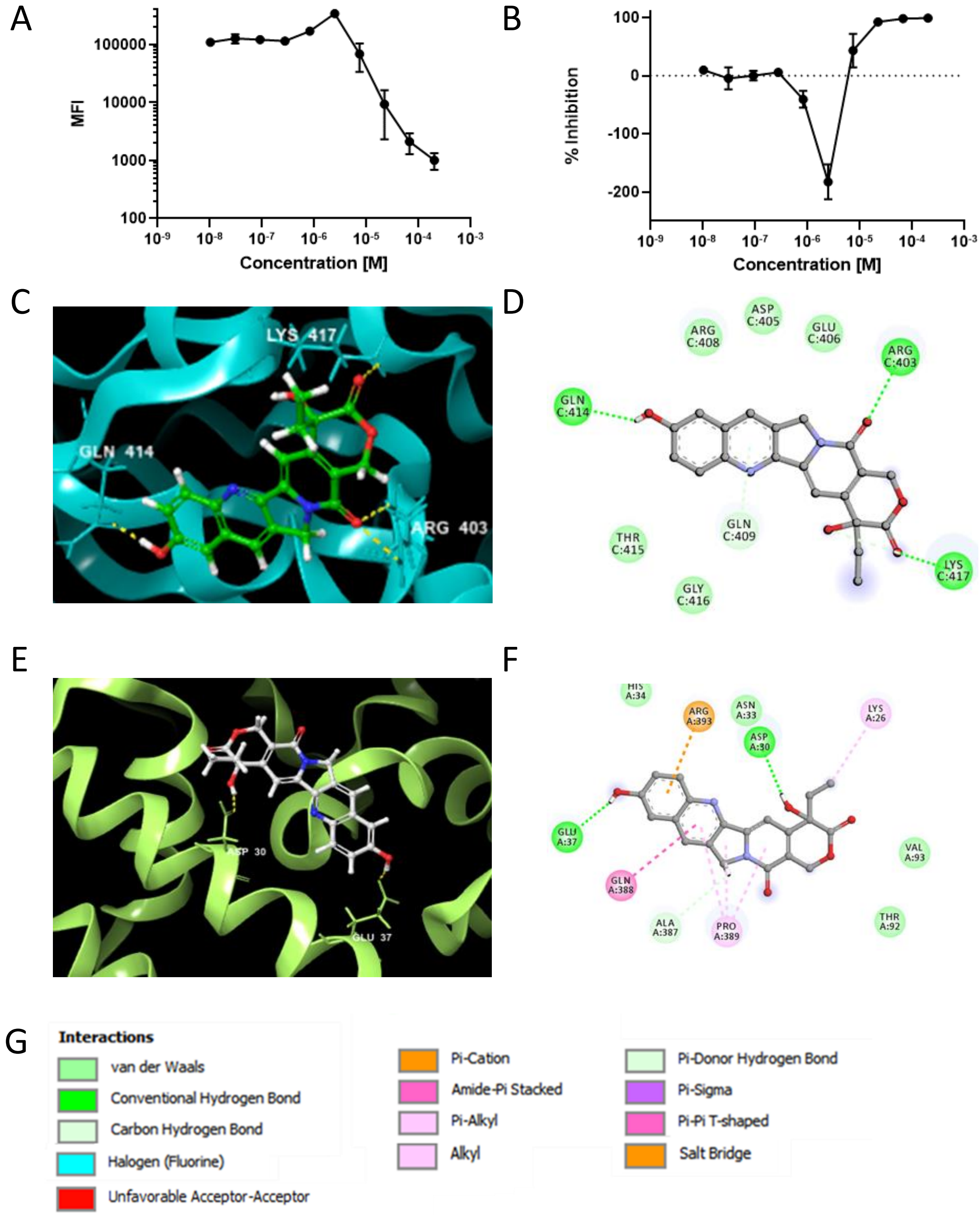

Figure S5: EC50 and computational binding data for Hydroxy Camptothecine. A,B) EC50 data are expressed as median fluorescent intensity (MFI, A) or percent inhibition (B). C) Three dimensional and D) two dimensional computationally determined lowest-energy docking poses of Hydroxy Camptothecine and the SARS-CoV-2 spike protein. E) Three dimensional and F) two dimensional computational docking of Hydroxy Camptothecine and human ACE2. G) Key showing color-codes for interaction types.

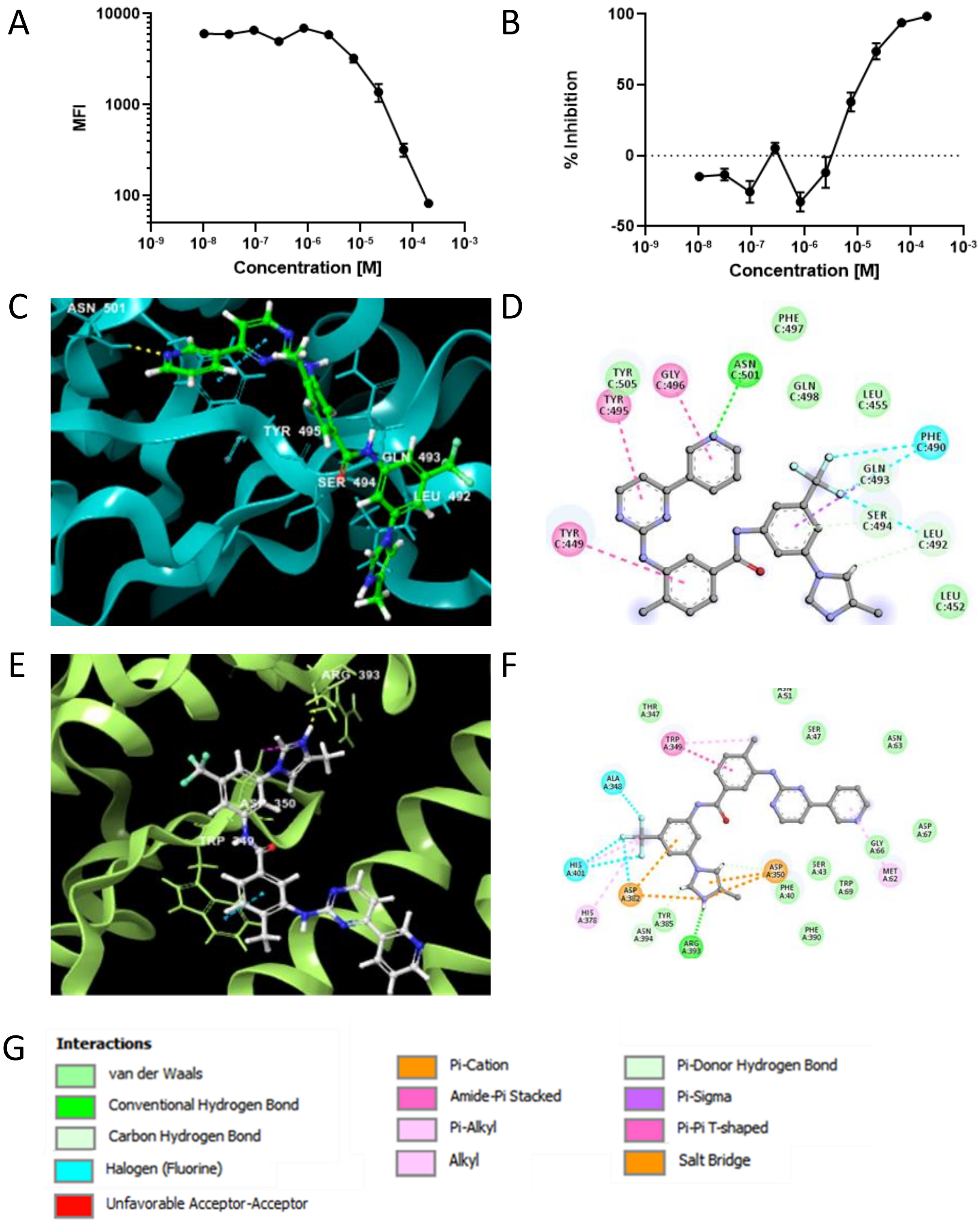

Figure S6: EC50 and computational binding data for Nilotinib HCl. A,B) EC50 data are expressed as median fluorescent intensity (MFI, A) or percent inhibition (B). C) Three dimensional and D) two dimensional computationally determined lowest-energy docking poses of Nilotinib HCl and the SARS-CoV-2 spike protein. E) Three dimensional and F) two dimensional computational docking of Nilotinib HCl and human ACE2. G) Key showing color-codes for interaction types.

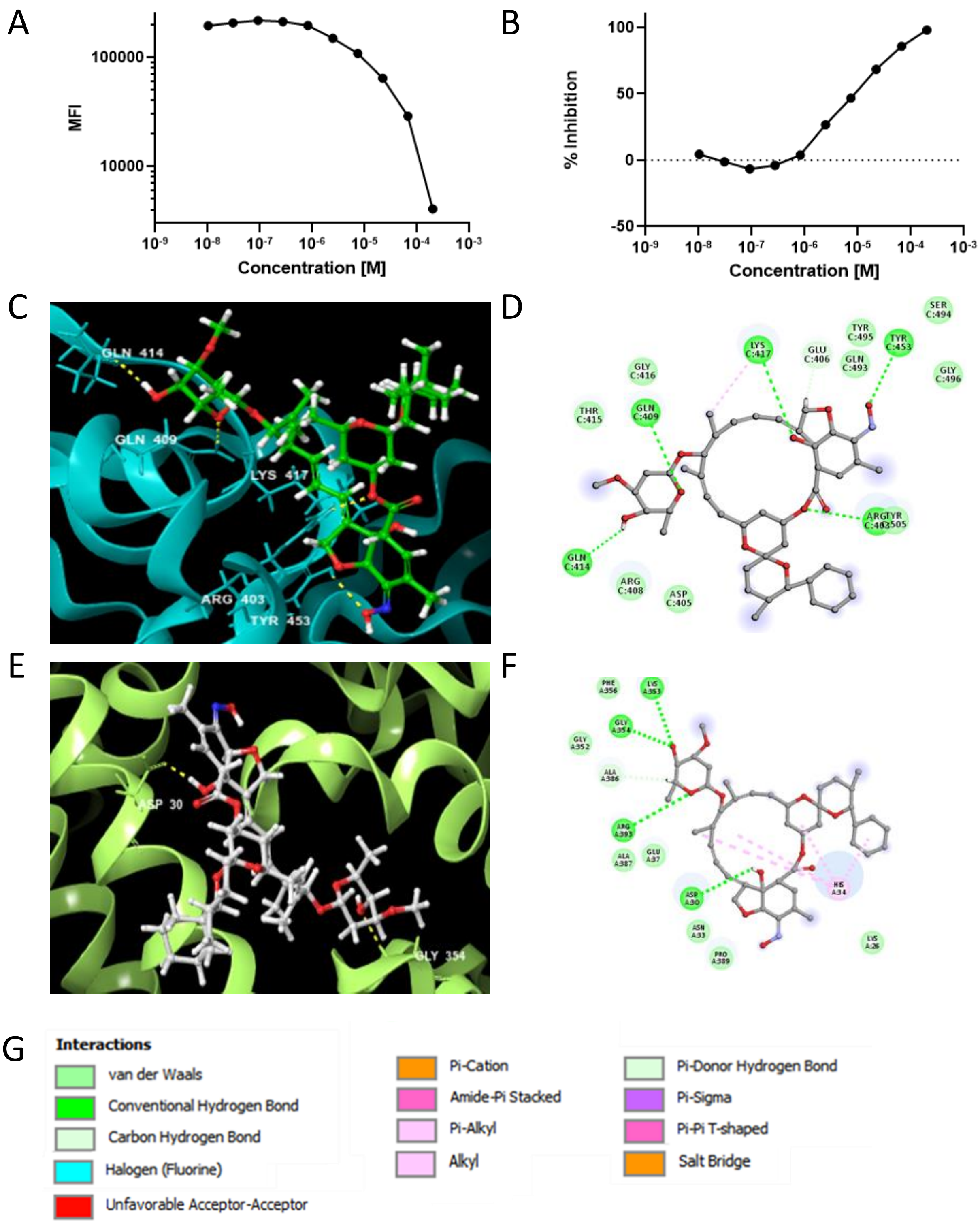

Figure S7: EC50 and computational binding data for Selamectin. A,B) EC50 data are expressed as median fluorescent intensity (MFI, A) or percent inhibition (B). C) Three dimensional and D) two dimensional computationally determined lowest-energy docking poses of Selamectin and the SARS-CoV-2 spike protein. E) Three dimensional and F) two dimensional computational docking of Selamectin and human ACE2. G) Key showing color-codes for interaction types.

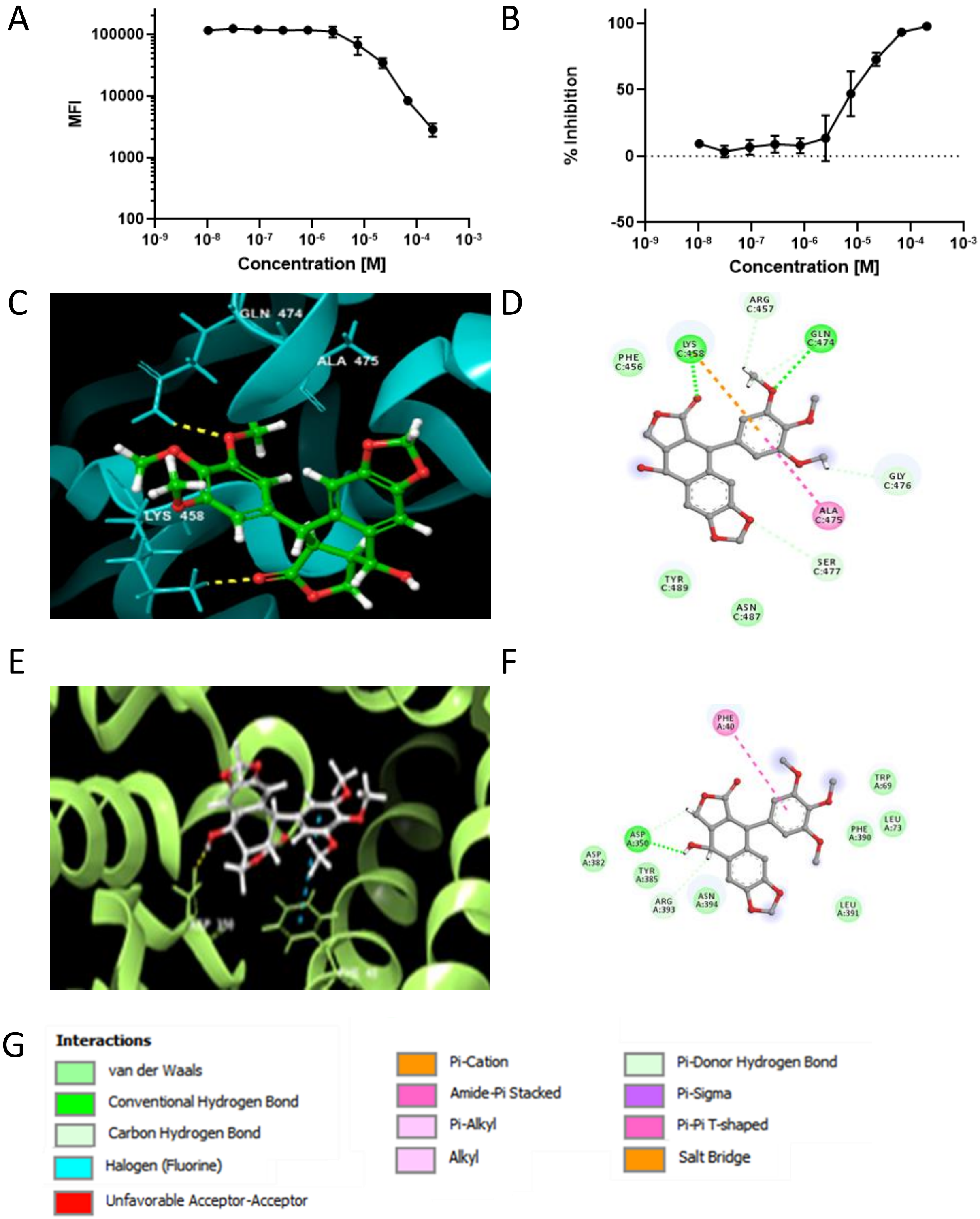

Figure S8: EC50 and computational binding data for Picropodophyllin. A,B) EC50 data are expressed as median fluorescent intensity (MFI, A) or percent inhibition (B). C Three dimensional and D) two dimensional computationally determined lowest-energy docking poses of Picropodophyllin and the SARS-CoV-2 spike protein. E) Three dimensional and F) two dimensional computational docking of Picropodophyllin and human ACE2. G) Key showing color-codes for interaction types.

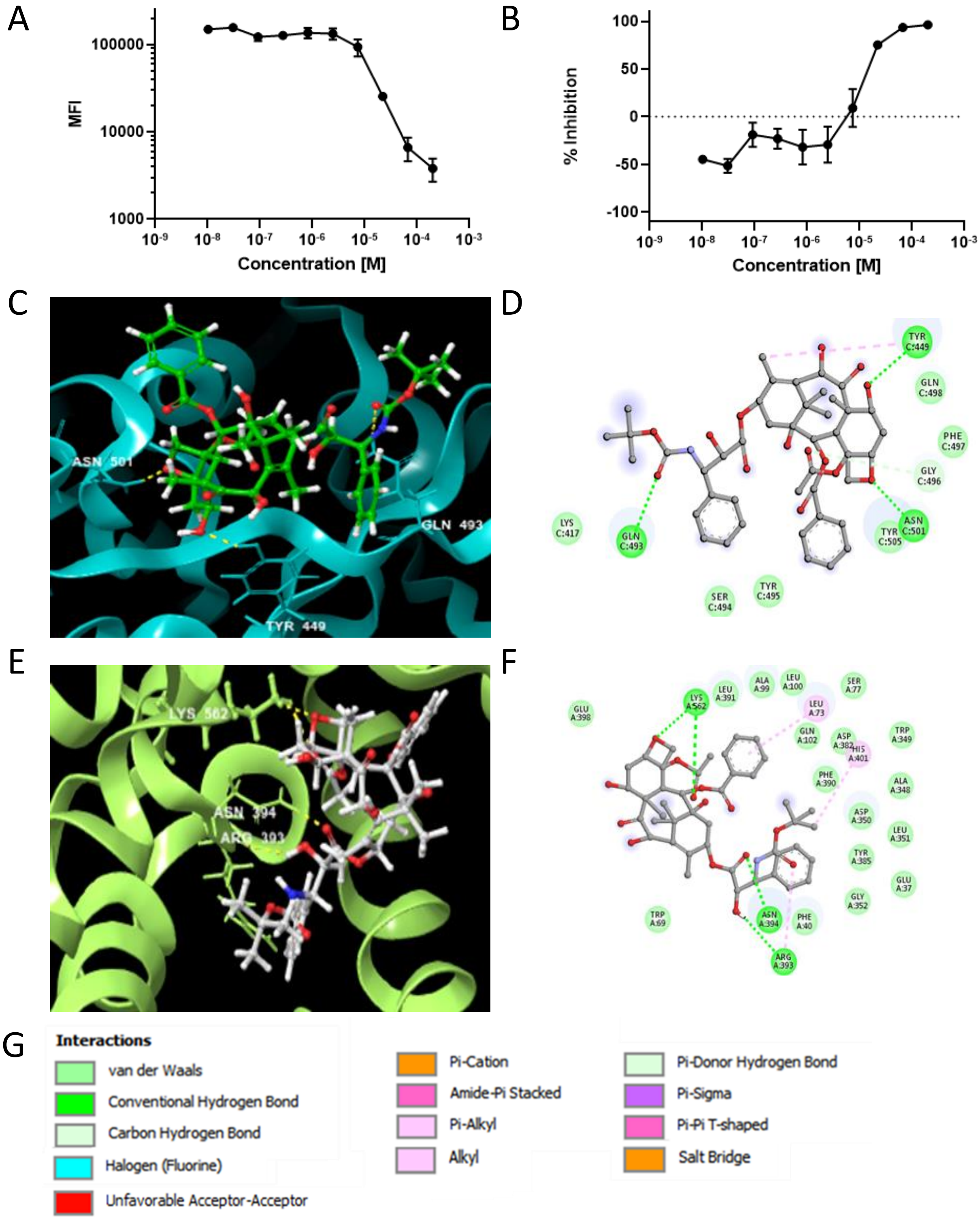

Figure S9: EC50 and computational binding data for Docetaxel. A,B) EC50 data are expressed as median fluorescent intensity (MFI, A) or percent inhibition (B). C) Three dimensional and D) two dimensional computationally determined lowest-energy docking poses of Docetaxel and the SARS-CoV-2 spike protein. E) Three dimensional and F) two dimensional computational docking of Docetaxel and human ACE2. G) Key showing color-codes for interaction types.

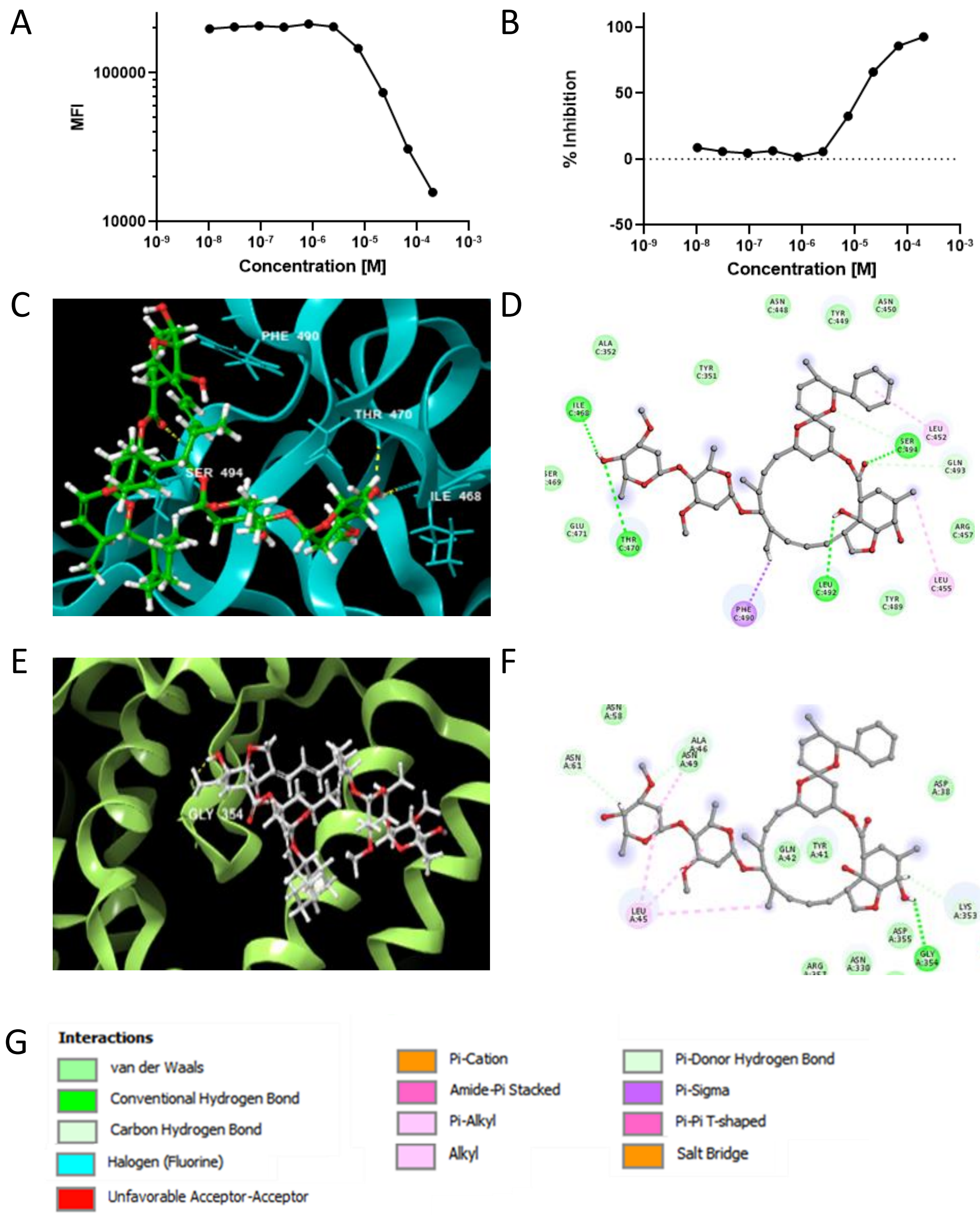

Figure S10: EC50 and computational binding data for Doramectin. A,B) EC50 data are expressed as median fluorescent intensity (MFI, A) or percent inhibition (B). C) Three dimensional and D) two dimensional computationally determined lowest-energy docking poses of Doramectin and the SARS-CoV-2 spike protein. E) Three dimensional and F) two dimensional computational docking of Doramectin and human ACE2. G) Key showing color-codes for interaction types.

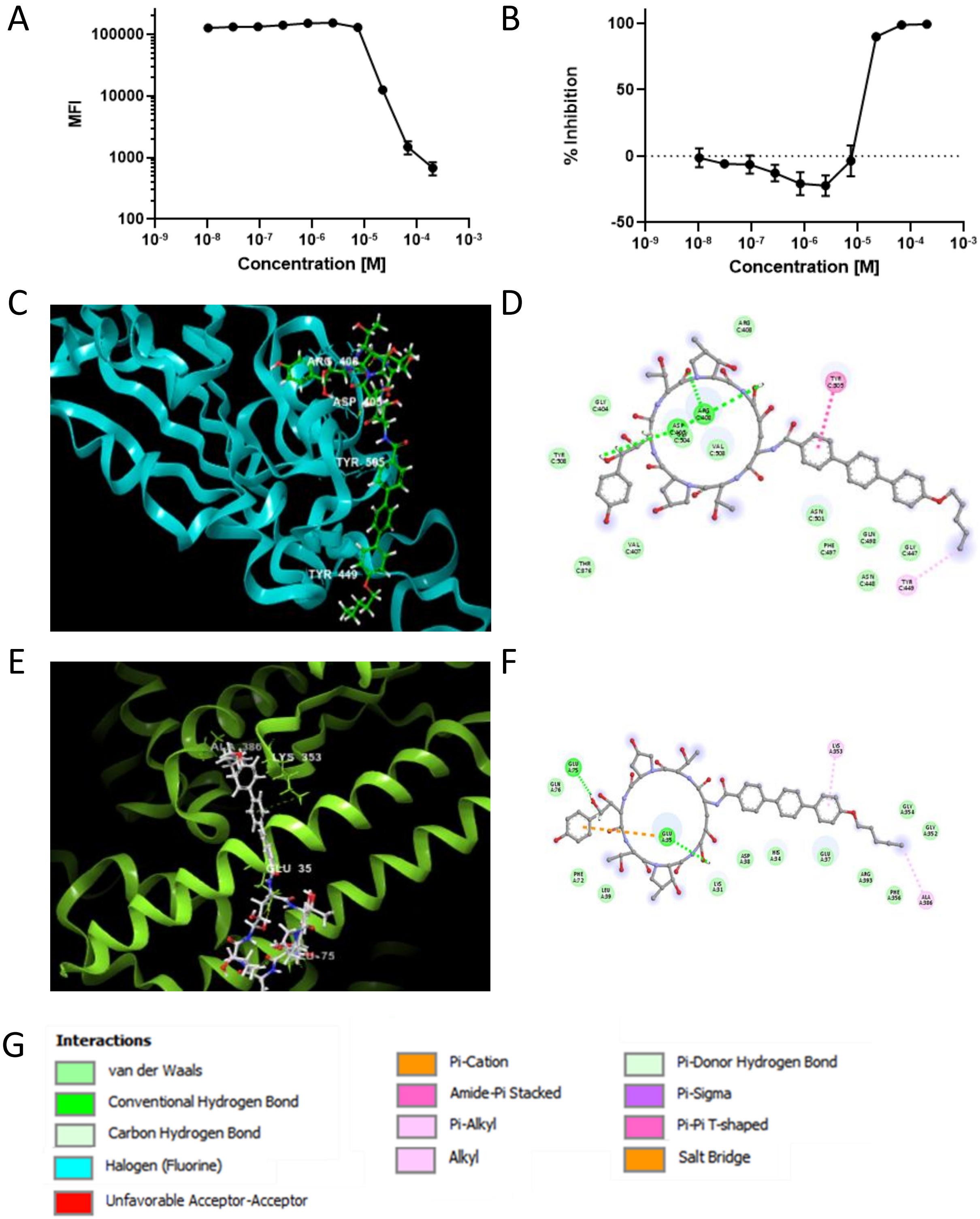

Figure S11: EC50 and computational binding data for Anidulafungin. A,B) EC50 data are expressed as median fluorescent intensity (MFI, A) or percent inhibition (B). C) Three dimensional and D) two dimensional computationally determined lowest-energy docking poses of Anidulafungin and the SARS-CoV-2 spike protein. E) Three dimensional and F) two dimensional computational docking of Anidulafungin and human ACE2. G) Key showing color-codes for interaction types.

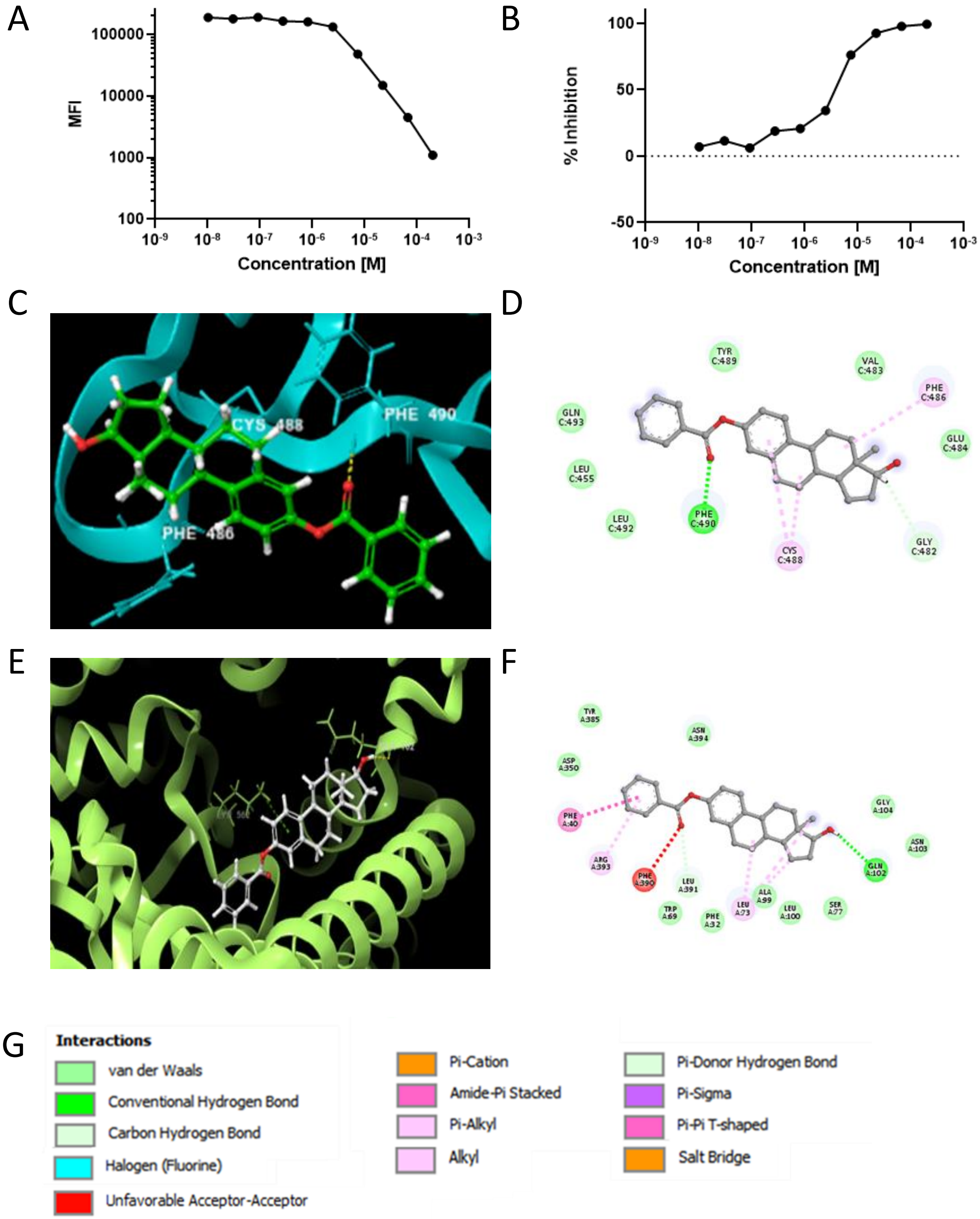

Figure S12: EC50 and computational binding data for Estradiol Benzoate.

A,B) EC50 data are expressed as median fluorescent intensity (MFI, A) or percent inhibition (B). C) Three dimensional and D) two dimensional computationally determined lowest-energy docking poses of Estradiol Benzoate and the SARS-CoV-2 spike protein. E) Three dimensional and F) two dimensional computational docking of Estradiol Benzoate and human ACE2. G) Key showing color-codes for interaction types.
